# Spatiotemporal trends and socioecological factors associated with Lyme disease in eastern Ontario, Canada from 2010–2017

**DOI:** 10.1186/s12889-022-13167-z

**Published:** 2022-04-13

**Authors:** Andreea M. Slatculescu, Claudia Duguay, Nicholas H. Ogden, Beate Sander, Marc Desjardins, D. William Cameron, Manisha A. Kulkarni

**Affiliations:** 1 School of Epidemiology and Public Health, Faculty of Medicine, University of Ottawa, 600 Peter Morand Crescent, Ottawa, ON K1G 5Z3 Canada; 2 Public Health Risk Sciences Division, National Microbiology Laboratory, Public Health Agency of Canada, Saint-Hyacinthe, QC Canada; 3 Toronto Health Economics and Technology Assessment Collaborative, University Health Network, Toronto, ON Canada; 4 Institute of Health Policy, Management and Evaluation, University of Toronto, Toronto, ON Canada; 5 Public Health Ontario, Toronto, ON Canada; 6 ICES, Toronto, ON Canada; 7 Department of Pathology and Laboratory Medicine, University of Ottawa, Ottawa, ON Canada; 8 Division of Microbiology, Eastern Ontario Regional Laboratory Association, Ottawa, ON Canada; 9 Department of Biochemistry, Microbiology and Immunology, Faculty of Medicine, University of Ottawa, Ottawa, ON Canada; 10 Chronic Disease Program, Ottawa Hospital Research Institute, Ottawa, ON Canada; 11 Department of Medicine, Faculty of Medicine, University of Ottawa, Ottawa, ON Canada

## Abstract

Currently, there is limited knowledge about socioeconomic, neighbourhood, and local ecological factors that contribute to the growing Lyme disease incidence in the province of Ontario, Canada. In this study, we sought to identify these factors that play an important role at the local scale, where people are encountering ticks in their communities. We used reported human Lyme disease case data and tick surveillance data submitted by the public from 2010–2017 to analyze trends in tick exposure, spatiotemporal clusters of infection using the spatial scan statistic and Local Moran’s I statistic, and socioecological risk factors for Lyme disease using a multivariable negative binomial regression model. Data were analyzed at the smallest geographic unit, consisting of 400–700 individuals, for which census data are disseminated in Canada. We found significant heterogeneity in tick exposure patterns based on location of residence, with 65.2% of Lyme disease patients from the city of Ottawa reporting tick exposures outside their health unit of residence, compared to 86.1%—98.1% of patients from other, largely rural, health units, reporting peri-domestic exposures. We detected eight spatiotemporal clusters of human Lyme disease incidence in eastern Ontario, overlapping with three clusters of *Borrelia burgdorferi*-infected ticks. When adjusting for population counts, Lyme disease case counts increased with larger numbers of *Borrelia burgdorferi*-infected ticks submitted by the public, higher proportion of treed landcover, lower neighbourhood walkability due to fewer intersections, dwellings, and points of interest, as well as with regions of higher residential instability and lower ethnic concentration (Relative Risk [RR] = 1.25, 1.02, 0.67–0.04, 1.34, and 0.57, respectively, p < .0001). Our study shows that there are regional differences in tick exposure patterns in eastern Ontario and that multiple socioecological factors contribute to Lyme disease risk in this region.

**Supplementary Information:**

The online version contains supplementary material available at 10.1186/s12889-022-13167-z.

## Background

Lyme disease is a tick-borne illness caused, in northeastern and midwestern North America, by infection with the bacterium *Borrelia burgdorferi* sensu stricto, which is transmitted through the bite of the blacklegged tick, *Ixodes scapularis* [1, 2]. Lyme disease is the most prevalent vector-borne illness in North America and, with the northward spread of ticks from endemic regions in the United States and southern Canada, its incidence has also increased substantially in central and eastern Canada [3–8]. The continued range expansion of *I. scapularis* is in part attributed to a warming climate increasing environmental suitability for tick establishment, permitting *B. burgdorferi* transmission cycles to establish between wildlife hosts and reproducing tick populations [9–14]. Ecologically, *I. scapularis* colonization depends on a complex set of factors including the presence of deciduous forests, shrubs, and forest understory, increased ground temperature and degree-days above 0 °C, and a higher proportion of forest fragmentation that favours the abundance of animal hosts such as white-tailed deer and white-footed mice [15–19].

There is evidence that human infection with Lyme disease in regions where blacklegged ticks are established is correlated with the density of host-seeking *I. scapularis* and the prevalence of *B. burgdorferi* infection in ticks, although there is heterogeneity at different spatial scales [20–23]. Recent studies by Ripoche et al. (2018) in Quebec and Gasmi et al. (2019) in Ontario and Manitoba showed that the cumulative number of locally acquired *I. scapularis* ticks submitted by the general public to local health units was a strong indicator of municipalities at risk of Lyme disease [24, 25]. However, the extent to which ecological risk factors influence the risk of contracting Lyme disease also depends on factors that increase human exposure and accessibility to risk areas, such as location of residence, type of occupation, where people undertake leisure activities, and overall knowledge, attitudes, and practices relating to tick exposures and preventive measures [26–28]. Recently, Bouchard et al. (2018) developed an integrated social-behavioural and ecological risk map to characterize multiple components of Lyme disease risk in the Montérégie region of southern Quebec, and while individual social-behavioural variables were not significantly associated with Lyme disease cases, they found that social-behavioural variables had a markedly different distribution compared to ecological variables, indicating that various factors may have different impacts on human Lyme disease infection [29].

In Ontario, most studies have focused on the distribution of the blacklegged tick vector as a measure of environmental risk for Lyme disease and on ecological factors that contribute to tick establishment and northward spread [9, 10, 17, 30]. However, little is known about how neighbourhood structure and other socioecological factors affect human exposure to these environmental risk areas. Tick exposure location for Lyme disease patients is also often approximated to broad geographic regions or assumed to be near the home residence, further highlighting the need for fine-scale studies and more accurate analysis of tick exposure [7, 24, 25, 31]. In this study, we use human Lyme disease case data and passive tick surveillance data, consisting of ticks submitted by the public to local public health units (PHUs), to analyze tick exposure patterns for Lyme disease patients and tick submitters as well as spatiotemporal trends in Lyme disease incidence and socioecological risk factors at the smallest geographic unit for which census data are available in Canada, the dissemination area (DA). DAs are defined as small, relatively stable geographic units with a population of 400 to 700 persons bounded by features such as roads, railways, and water sources [32].

## Methods

### Study area

**Fig.1:**
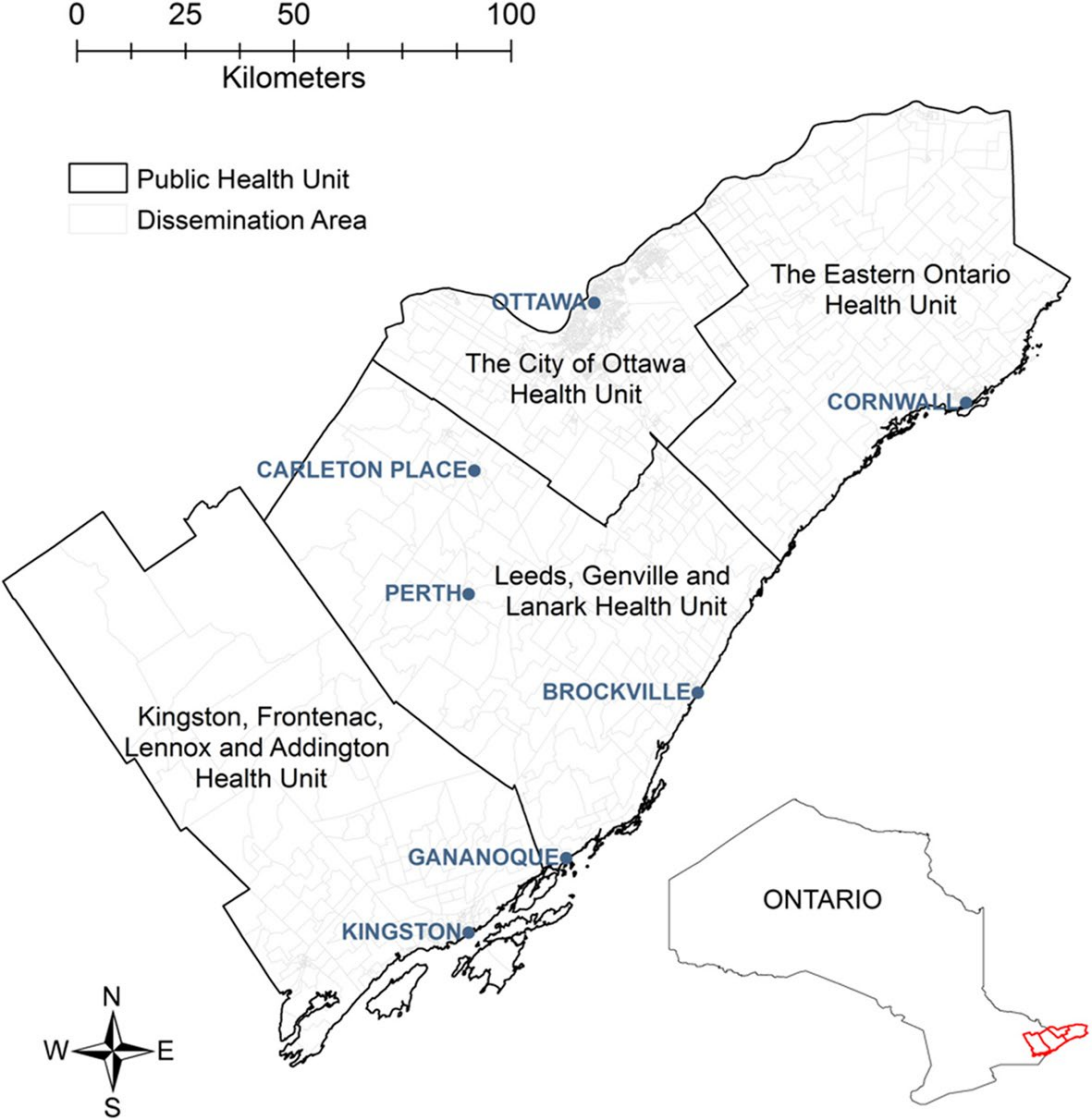
Map showing the study area and inset showing the location of the four health units in the province of Ontario, Canada

Our study encompassed four PHUs in eastern Ontario, Canada: Eastern Ontario Health Unit (EOH), City of Ottawa Health Unit (OTT), Leeds, Grenville, and Lanark Health Unit (LGL), and Kingston, Frontenac, Lennox and Addington Health Unit (KFL) (Fig. 1). The region is largely rural with several population centres including Ottawa (population 934,243), Kingston (population 123,798), and Cornwall (population 46,589) [33].

### Surveillance data

We obtained reported human Lyme disease case data (2010–2017) from the Integrated Public Health Information System (iPHIS) database, which contains patient information and laboratory test results for reportable diseases in Ontario. We defined cases as patients with confirmed or probable Lyme disease according to the national case definition for Lyme disease (see Additional file 1) [34]. We also obtained passive tick surveillance data (2010–2017) from Public Health Ontario (PHO), which receives and identifies ticks from PHUs and healthcare providers and collates these data with real-time polymerase chain reaction (PCR) test results from the National Microbiology Laboratory. We retained all information available on patient and tick submitter home residence and self-reported travel history or location of tick exposure. For tick submissions, we retained *I. scapularis* specimens along with tick life stage and PCR test results for *B. burgdorferi*.

### Exposure analysis

We geocoded the home location and the most probable tick exposure location using a Google Application Programming Interface in R v.3.5.1 [35]. We defined the most probable tick exposure location as 1) the only exposure provided, or 2) the finest-scale location if multiple fields were provided and the locations were reasonably close (e.g., trail versus neighbourhood). If multiple locations with no geographic overlap were reported, we removed the case or tick from analysis. We then linked home and exposure locations with the DA geographic boundaries obtained from the 2016 Canada Census (Statistics Canada) using ArcGIS v.10.5.1 (ESRI, Redlands, CA, USA). For those with a broader exposure location that could not be easily geocoded (e.g., city/suburb level), we assigned the exposure to the nearest census division and used a weighted approach to subsequently link each data point to a DA. The weight was derived from a maximum entropy species distribution model for *I. scapularis* developed by Slatculescu et al. (2020) and represents the average predicted habitat suitability for *I. scapularis* per DA; thus, allowing the exposure to be assigned to a biologically plausible location within the region indicated by the individual [10]. For patients that provided additional notes, we created a separate variable classifying the type of exposure (e.g., occupational, recreational, cottage, and home/yard). We used frequency tables to identify characteristics of tick submitters and Lyme disease patients, tick exposure locations compared to home residence, and types of exposures. We used the Chi-Square test (or the Fisher’s exact test for cells with fewer than 5 observations) and the two-sided t test (α = 0.05) to determine if there was a relationship between categorical variables or to compare the means of two independent groups, respectively. Statistical analyses were conducted using SAS v.9.4 (SAS Institute, Cary, NC, USA).

### Cluster analysis

We used SaTScan™ software (v 9.4.1 Kulldorff and Information Management Services, Inc.) to detect spatiotemporal clusters of human Lyme disease cases and infected ticks in eastern Ontario [36]. This method uses a cylindrical scanning window across all spatial and temporal locations to identify regions where the observed numbers of cases or ticks exceed the expected numbers in a comparison region under the null hypothesis of spatial randomness [37]. To detect clusters of human Lyme disease cases, we used a Poisson-based probability model, with patient and census population counts per DA. To detect clusters of infected ticks, we used a Bernoulli-based probability model, with the outcome being ticks testing positive and negative for *B. burgdorferi*. Since we only had DA-level exposure for most individuals, we analyzed clusters at the DA by assigning cases and ticks the centroid coordinates for the DA in which they were exposed. Therefore, cluster size is based on the number of DAs with higher observed counts than expected counts (i.e., a cluster radius of 0 km indicates high counts in a single DA). We did not permit spatial overlap between clusters, we used a one-year minimum temporal window, and 5% or 30% maximum spatial cluster sizes for cases and ticks, respectively, were set a priori to account for the low infection rates [38]. The most likely clusters were detected using a likelihood ratio test and p-values were calculated based on maximum likelihood rank using 999 Monte Carlo replications (*p* < 0.05 was considered significant).

We used ArcGIS v10.5.1 to generate Anselin’s Local Moran’s I statistic to better characterize clusters based on spatial autocorrelation among neighbouring DAs, under the null hypothesis that there is no association between values of human Lyme disease or infected ticks in nearby DAs [39]. Statistical significance was determined using Z-scores (α = 0.05). A positive value for I indicates that a DA has neighbouring DAs with similarly high or low infection rates; hence, the DA is part of a cluster. A negative value for I indicates that a DA has neighbouring DAs with dissimilar infection rates; hence the DA is an outlier. The cluster/outlier types were categorized as statistically significant cluster of high infection (high-high), cluster of low infection (low-low), outlier of high infection surrounded by primarily low infection (high-low), and outlier of low infection surround by primarily high infection (low–high). Since we are interested in detecting regions with high Lyme disease incidence and tick infection rates, we only mapped high-high clusters and high-low outliers.

### Risk factor analysis

We constructed a multivariable regression model to assess the relationship between the outcome variable of DA-level number of human Lyme disease cases and multiple socioecological variables, defined in Additional file 1. All variables were obtained or calculated at the DA level. Lyme disease case counts and publicly submitted ticks from 2010–2017 were aggregated per DA. Ecological variables of interest included: numbers of publicly submitted *I. scapularis* ticks and nymphs, total positive *B. burgdorferi* ticks, proportion of mixed treed land and infrastructure derived from the Southern Ontario Land Resource Information System (SOLRISv3.0) from the Ministry of Natural Resources and Forestry’s open data portal (https://geohub.lio.gov.on.ca), and neighbourhood walkability score obtained from the Canadian Active Living Environments (Can-ALE) database from McGill University (https://nancyrossresearchgroup.ca/research/can-ale/). Socioeconomic variables of interest included median income, commute to work duration, language knowledge, and population density obtained from the 2016 Canada Census (Statistics Canada), as well as the 2016 Ontario Marginalization Index (ON-Marg) available from PHO. The ON-Marg was derived from a series of iterative factor analyses of 42 census-based indicators, yielding four factors with 18 remaining indicators that represent multiple dimensions of socioeconomic status in Ontario [40]. These factors are 1) Residential instability: a measure of high rates of family and housing instability (indicators are proportion of population who is living alone, who is not youth age 5–15, who is single/widowed/divorced and who moved during the last 5 years, and dwellings that are not owned or that are apartments), 2) Material deprivation: a measure of inability to access and attain basic material needs (indicators are proportion of population who does not have a high school diploma, who are unemployed, who are considered low income, who are single parent families, who receive government transfer payments, and whose housing is in need of major repair), 3) Dependency: a measure of people who do not have income from employment (indicators are proportion of population who is 65 years or older, who are not participating in the labour force, and a high dependency ratio of youth (0–14 years) and elderly (65 + years) per working population 15–64 years), and 4) Ethnic concentration: a measure of recent immigrants and visible minorities (indicators are proportion of population who arrived in Canada in the last 5 years and persons, other than Indigenous peoples, who are non-Caucasian in race or non-white in colour) [40]. The ON-Marg is available at a fine spatial scale (DA-level) and was selected to analyze multiple aspects of the broader socioeconomic risk factors for LD.

We used an extended Poisson regression with a negative binomial response for the outcome to account for overdispersion of Lyme disease cases per DA. Model fit was assessed by visual inspection of residuals and the deviance value. Each predictor variable was analyzed individually and only those significantly (*p* < 0.05) associated with Lyme disease counts were considered in the multivariable model. We also tested for correlation among potential predictors and retained those with low to moderate correlation (|Pearson’s r|≤ 0.6). We further included the natural log of human population size as a predictor since we expect number of tick submissions and Lyme disease cases to increase with population. We selected the final model based on the Akaike Information Criterion (AIC) when compared to smaller nested models.

### Sensitivity analysis

We conducted a sensitivity analysis to verify the validity of our weighted approach for tick exposure. We repeated all analyses with a smaller subset of patient (*n* = 589) and tick (*n* = 1,684) data that had geographically precise exposure locations and compared this with our global analyses that also included weighted exposures by DA-habitat suitability for *I. scapularis* for broader exposure locations. Results from the sensitivity analysis are presented in the supplemental materials.

## Results

### Trends in human Lyme disease cases and tick exposures in eastern Ontario

**Fig. 2:**
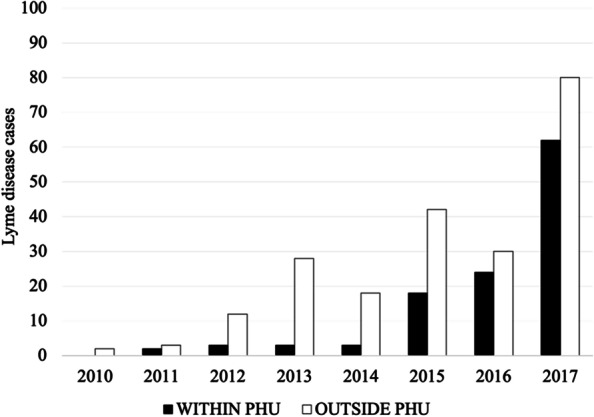
Annual Lyme disease cases and tick submissions with exposure within and outside the City of Ottawa Health Unit (OTT) from 2010–2017

There were 1,224 Lyme disease cases and 6,706 *I. scapularis* ticks acquired in Ontario between 2010–2017 among residents of our study area. We found that most tick exposures occurred within the PHU of residence, with OTT Lyme disease patients being the notable exception (Table 1). In OTT, 65.2% (*n* = 215) of Lyme disease patients and 26.8% (*n* = 404) of tick submitters reported tick exposures outside their health unit of residence compared to 13.9% (*n* = 10), 1.9% (*n* = 8), and 4.0% (*n* = 16) of Lyme disease patients and 11.1% (*n* = 71), 8.1% (*n* = 226), and 19.2% (*n* = 337) of tick submitters for EOH, LGL, and KFL, respectively (Table 1). Despite the greater number of outside-PHU tick exposure for OTT Lyme disease patients, within-PHU tick exposures also increased annually, indicating a growing risk at home and via travel-related exposures (Fig. 2).

**Table 1:** Lyme disease cases and public tick submissions with exposure within and outside the public health unit of residence

	Exposure within PHU	Exposure outside PHU	*P* value
Lyme disease cases, n (%)			
EOH	62 (86.1)	10 (13.9)	< .0001
OTT	115 (34.8)	215 (65.2)	< .0001
LGL	416 (98.1)	8 (1.9)	< .0001
KFL	382 (96.0)	16 (4.0)	< .0001
Tick submissions, n (%)			
EOH	567 (88.9)	71 (11.1)	< .0001
OTT	1105 (73.2)	404 (26.8)	< .0001
LGL	2580 (91.9)	226 (8.1)	< .0001
KFL	1416 (80.8)	337 (19.2)	< .0001

*PHU* Public health unit, *EOH* Eastern Ontario Health Unit, *OTT* City of Ottawa Health Unit, *LGL* Leeds, Grenville, and Lanark Health Unit, *KFL* Kingston, Frontenac, Lennox and Addington Health Unit

For patients who provided extra exposure notes, home/yard was most reported by patients from EOH (*n* = 36) and LGL (*n* = 186), whereas OTT patients reported a split between cottage exposure (*n* = 81) and home/yard (*n* = 70) as well as recreational activities (*n* = 25) and occupational/school exposures (*n* = 21). The cottage exposures indicated by OTT patients were mostly attributed to locations in LGL (*n* = 69), KFL (*n* = 23), and other PHUs outside our study area (*n* = 6). For KFL patients, this type of exposure information was largely unavailable because most cases were attributed to unspecified local tick exposure due to the high endemicity of *I. scapularis* in this region.

### Spatiotemporal clusters of human Lyme disease cases and infected ticks in eastern Ontario

**Fig. 3:**
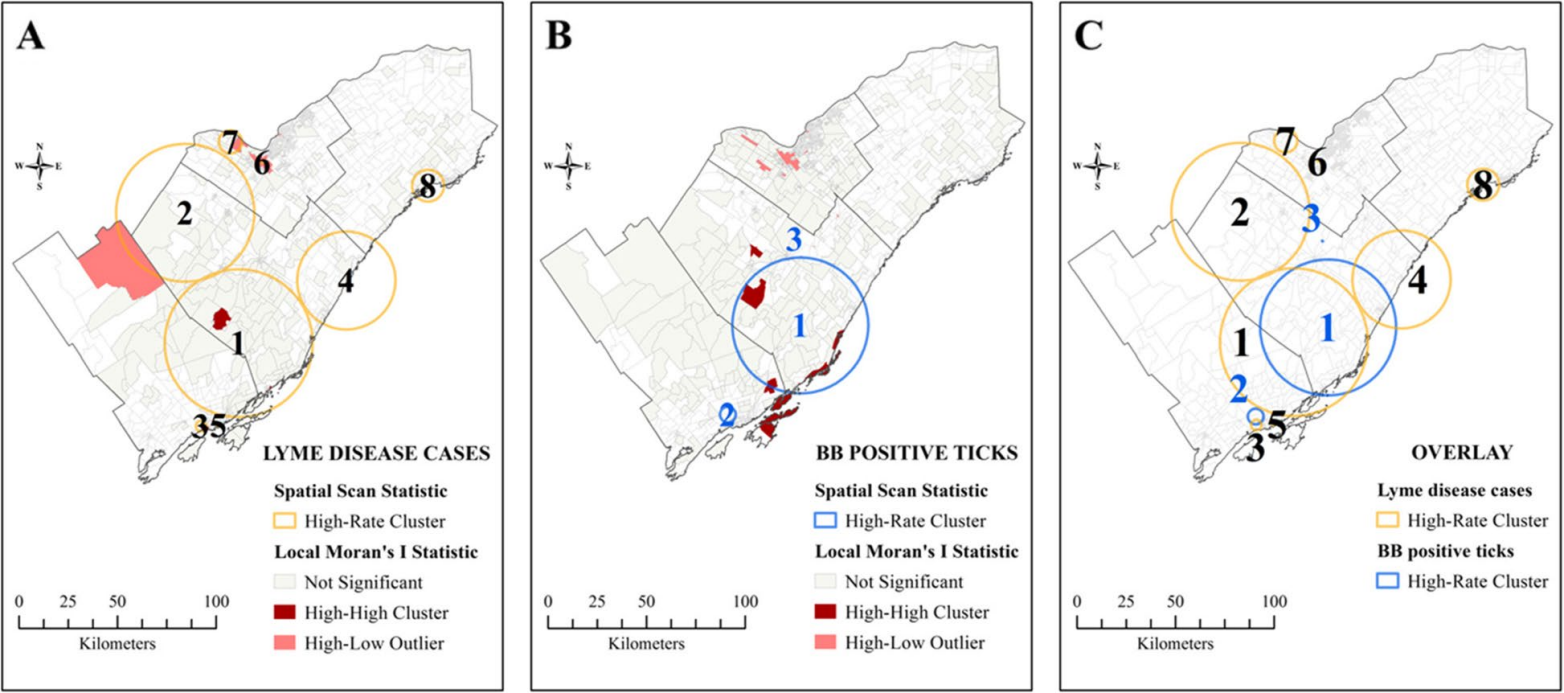
Spatiotemporal clusters of Lyme disease (**A**) and Borrelia burgdorferi infected ticks (**B**) in eastern Ontario, Canada, from 2020–2017. The overlay of the two shows geographical overlap between clusters (**C**)

We detected eight statistically significant spatiotemporal clusters of human Lyme disease cases (Fig. 3). The three largest clusters were located across LGL (Cluster 1: radius 36.7 km, years 2014–2017, relative risk [RR] = 19.09, *p* < 0.0001; Cluster 2: 34.4 km, 2017, RR = 15.02, *p* < 0.0001; Cluster 4: 24.4 km, 2015–2017, RR = 5.13, *p* < 0.0001), two smaller clusters located in KFL (Cluster 3: 2.6 km, 2017, RR = 38.57, *p* < 0.0001; Cluster 5: 0.4 km, 2017, RR = 112.97, *p* < 0.0001) and OTT (Cluster 6: 0 km, 2016–2017, RR = 82.85, *p* < 0.0001; Cluster 7: 5.8 km, 2015–2017, RR = 15.02, *p* < 0.0001) and one cluster in EOH (Cluster 8: 7.9 km, 2012–2015, RR = 6.44, *p* = 0.0002). The Local Moran’s I statistic detected statistically significant high-high clustering in LGL and KFL, indicating that neighbouring DAs have similarly high infection rates and Lyme disease risk is more geographically dispersed in these regions. In contrast, high-low clustering was detected in OTT, which suggests outlier DAs with high Lyme disease rates and more localized risk in the western region of Ottawa.

We also detected three statistically significant clusters of *B. burgdorferi*-infected ticks (Fig. 3). Two clusters were in LGL (Cluster 1: 33.7 km, 2012–2015, RR = 1.45, *p* < 0.0001; Cluster 3: 0 km, 2013–2016, RR = 3.60, *p* = 0.0490) and one in KFL (Cluster 2: 3.9 km, 2012–2013, RR = 2.25, *p* = 0.0022). The Local Moran’s I statistic detected a similar pattern as for human Lyme disease cases, with statistically significant high-high clustering in LGL and KFL and high-low outliers in OTT. Clusters of *B. burgdorferi*-infected ticks overlapped geographically with clusters of human Lyme disease cases in these regions, though most clusters of *B. burgdorferi*-infected ticks preceded those of human Lyme disease cases by 3–4 years.

### Socioecological risk factors for human Lyme disease infection

Lyme disease case counts per DA increased by approximately 25% for every unit increase in the number of *B. burgdorferi*-positive ticks submitted through passive tick surveillance (RR = 1.25, 95% CI = 1.19,1.31, *p* < 0.0001). Lyme disease counts also rose significantly with increased proportion of mixed treed land cover (RR = 1.02; 95% CI = 1.01,1.02; *p* = 0.0001) and with various neighbourhood characteristics associated with increased rurality (residential instability: RR = 1.36, 95% CI = 1.19,1.56, *p* < 0.0001; ethnic concentration: RR = 0.57, 95% CI = 0.45,0.73, *p* < 0.0001). In contrast, Lyme disease cases counts decreased with higher neighbourhood walkability score, which is associated with increased urbanization (Table 2).

**Table 2:** Negative binomial regression model showing socioecological factors associated with Lyme disease case counts in eastern Ontario, Canada from 2010–2017

Parameter	RR	95% CI (lower)	95% CI (upper)	*P* value
Intercept	0.0232	0.0049	0.109	< .0001
*B. burgdorferi-*positive ticks	1.2464	1.1860	1.3098	< .0001
Walk score 1	1.0000	-	-	-
Walk score 2	0.6653	0.5068	0.8733	0.0033
Walk score 3	0.2106	0.1360	0.3261	< .0001
Walk score 4	0.0619	0.0177	0.2168	< .0001
Walk score 5	0.0478	0.0058	0.3949	0.0048
Proportion treed	1.0161	1.0079	1.0244	0.0001
Residential instability	1.3588	1.1853	1.5578	< .0001
Ethnic concentration	0.5744	0.4517	0.7304	< .0001
Log population	1.5851	1.2599	1.9941	< .0001

*RR* Relative risk, *CI* Confidence intervals

### Sensitivity analysis

For Lyme disease patients and tick submitters, particularly those from tick-endemic regions like KFL, home residence and year were strongly correlated with availability of exposure data, in part due to the lack of exposure-related data collected through disease and tick surveillance (see Additional file 2) [7, 31]. However, weighted exposures for Lyme disease cases and ticks showed a similar geographic pattern at the DA level compared to unweighted exposures despite the differences seen in the sources of the data (data not shown). We detected fewer clusters of Lyme disease and *B. burgdorferi*-infected ticks using the smaller dataset, although the larger patterns were consistent with our main analyses (see Additional file 3). We also found similar associations between Lyme disease case counts and socioecological predictors when weighted exposures were removed from analysis (see Additional file 4).

## Discussion

In this study, we found heterogeneity in tick exposure patterns by public health unit of residence. Lyme disease patients and residents of the three largely rural public health units, EOH, LGL, and KFL, reported tick exposure locations near the home, consistent with comparable studies in the United States that found high levels of peri-domestic tick exposures [41–43]. In contrast, individuals from the major population centre of Ottawa reported many tick exposures outside their health unit of residence as well as increasing annual exposures within the city, highlighting the heterogeneity in risk at finer spatial scales. A recent study by Tulloch et al. (2019) in the United Kingdom showed that Lyme disease incidence was associated with higher socioeconomic status (based on patients’ residence postcode) and with residence in more rural regions, suggesting that socioecological factors and neighbourhood characteristics may contribute to the marked difference between OTT and other PHUs [44].

We detected several spatiotemporal clusters of Lyme disease incidence and *B. burgdorferi*-infected ticks in LGL and KFL, with high spatial autocorrelation among DA infection rates. This pattern signals the presence of foci of tick establishment and subsequent expansion from these regions [45, 46]. We also detected a few smaller spatiotemporal clusters in OTT that, in contrast, showed low spatial autocorrelation among DA infection rates, indicating this region represents a newer area of tick establishment with more localized risk in outlier regions [14]. Our results differ somewhat from a recent study by Kulkarni et al. (2019) that found spatiotemporal clusters of Lyme disease and *B. burgdorferi-*infected ticks in KFL but not elsewhere in eastern Ontario [31]. These differences are likely due to several factors including the spatial scale at which clusters were investigated (i.e., smaller DA, ~ 20,000 total number in Ontario, versus the larger forward sortation area of the postal code, with ~ 500 total number in Ontario), the length of study period (i.e., we include an additional year with a large increase in number of cases), and exposure characteristics (i.e., we include travel-related exposures in nearby health units and used tick exposure location instead of home location).

Lyme disease infection in eastern Ontario increased with higher proportion of treed land, greater numbers of *B. burgdorferi*-positive ticks submitted by the public, and lower neighbourhood urbanity indicated by less favourable walking environments, lower ethnic concentration, and higher residential instability. These results are consistent with our knowledge regarding ecological risk for Lyme disease and provide evidence that tick densities at the community-level and local neighbourhood structure and characteristics are important determinants of Lyme disease risk [30, 31, 43]. The associations found in this study between human Lyme disease infection and area-level measures of marginalization provide the first evidence of socioeconomic risk factors for Lyme disease in the Canadian context. Factors of the ON-Marg such as ethnic concentration, a measure of recent immigrants and visible minorities (defined by Statistics Canada as persons, other than Indigenous people, who are non-Caucasian in race or non-White in colour), and residential instability, a measure of home security, home ownership and occupancy, represent multidimensional metrics of socioeconomic status, making it difficult to draw conclusions about the contribution of individual variables on Lyme disease risk [47]. In the United States, Springer and Johnson (2018) found evidence for comparable associations between Lyme disease infection and a relatively higher proportion of White population, higher average levels of education, lower poverty and crime rates, and increased vacant housing, of which 30% were seasonal or rental properties [48]. Together with our finding that most residents from the largely urban city of Ottawa were exposed to ticks outside of their health unit of residence, often citing cottage exposures, it is possible that the association with residential instability may be in part attributed to the higher rates of non-resident home ownership in recreational destinations such as Muskoka Lakes and Rideau Lakes [49]. However, further research would help to ascertain this mechanism of association and identify specific characteristics of neighbourhood structure that are driving the association between residential instability and ethnic concentration and Lyme disease risk in Ontario.

Our study has several limitations, the most significant being the sparsity of data pertaining to tick exposure locations collected via notifiable disease surveillance and passive tick surveillance data. We attempted to accurately identify tick exposure location at a fine geographic scale; however, to maximize data use for our analyses we used a weighted approach to assign most probable tick exposure locations when detailed data were unavailable. Our sensitivity analysis showed similar trends and results using a smaller fraction of data with detailed exposure, but we acknowledge that certain results such as our spatiotemporal cluster analyses may be influenced by the quality of data. Furthermore, due to regional differences in tick surveillance (i.e., cessation of passive tick surveillance submission from the public in endemic regions) we did not have complete data for publicly submitted ticks from LGL and KFL health units for later years of our study to explore additional temporal trends. Lastly, we used the four dimensions of the ON-Marg, derived from factor analyses of many census-based indicators, to assess the link more broadly between Lyme disease infection and socioeconomic risk factors. Additional research is warranted to better understand the nature of these relationships and potential public health implications. This study also focused on area-level socioecological risk factors and does not capture individual-level factors pertaining to home-ownership rates, household occupancy and structure, or potential factors such as under-reporting of Lyme disease by immigrants and minorities stemming from barriers accessing health care services or barriers to social integration and engaging in activities that increase exposure to Lyme disease (i.e., camping, outdoor sports and social interaction, cottages, etc.) that may also contribute to the association between Lyme disease risk and ethnic concentration or residential instability found in this study [50, 51]. On the other hand, our study has notable strengths as it represents the first analysis in Ontario, Canada looking at socioeconomic predictors of Lyme disease, which may help identify communities at higher risk of Lyme disease infection and to inform future studies looking at mechanisms of association and individual-level risk factors. Furthermore, we identified tick exposure location at the finest geographic unit possible and as a result we were able to detect heterogeneity in tick exposure patterns that would be overlooked at a larger spatial scale.

Our findings indicate that human Lyme disease risk is in part predicted by the spread of *I. scapularis* and *B. burgdorferi* and by various ecological and socioeconomic factors that affect access to environmental risk areas, with substantial heterogeneity in tick exposure patterns between rural and urban regions. This shows the importance of devising appropriate risk mitigation strategies and tailoring public health messaging to target different regions based on regional risk. Furthermore, it highlights the importance of ongoing Lyme disease surveillance methods and fine-scale studies to further identify neighbourhood-level patterns and determinants of Lyme disease and other tick-borne pathogens.

## Supplementary Information

**Additional file 1.** Definition of the outcome and predictor variables evaluated in the negative-binomial regression model looking at associations between Lyme disease (LD) prevalence and socioecological risk factors in eastern Ontario.

**Additional file 2.** Characteristics of Lyme disease cases and tick submitters with weighted versus unweighted tick exposure location.

**Additional file 3.** Sensitivity analysis of spatiotemporal clusters of Lyme disease (A) and *Borrelia burgdorferi* infected ticks (B) based on unweighted tick exposure location. The overlay of the two shows geographical overlap between clusters (C).

**Additional file 4.** Negative binomial regression model showing socioecological factors associated with Lyme disease case counts in eastern Ontario, Canada from 2010-2017 based on sensitivity analysis dataset with unweighted tick exposures.

## Data Availability

Tick surveillance data that support the findings of this study are available from Public Health Ontario (661 University Ave., Toronto, Ontario, M5G 1M1), but restrictions apply to the availability of these data, which were used under license for the current study, and so are not publicly available. These data are however available from the authors upon reasonable request and with permission of Public Health Ontario. Human Lyme disease case data are available from the City of Ottawa Public Health Unit (100 Constellation Dr., Nepean, Ontario, K2G 6J8), the Eastern Ontario Public Health Unit (872 rue Principale St., Casselman, Ontario, K0A 1M0), the Leeds, Grenville, and Lanark District Public Health Unit (458 Laurier Blvd., Brockville, Ontario, K6V 7A3), and the Kingston, Frontenac, and Lennox and Addington Public Health Unit (221 Portsmouth Ave., Kingston, Ontario, K7M 1V5), but restrictions apply to the availability of these data, which were used under license for the current study, and so are not publicly available. These data are however available from the authors upon reasonable request and with permission of each public health unit.
