# Supplementary material for: Spatiotemporal trends and socioecological factors associated with Lyme disease in eastern Ontario, Canada from 2010–2017
Source: BMC Public Health. 2022 Apr 13;22:736. doi: 10.1186/s12889-022-13167-z (PMC9006558; doi:10.1186/s12889-022-13167-z)
Supplement: Supplementary file 1 — Additional file 1. Definition of the outcome and predictor variables evaluated in the negative-binomial regression model looking at associations between Lyme disease (LD) prevalence and socioecological risk factors in eastern Ontario. [file 12889_2022_13167_MOESM1_ESM.docx]

Additional file 1. Definition of the outcome and predictor variables evaluated in the negative-binomial regression model looking at associations between Lyme disease (LD) prevalence and socioecological risk factors in eastern Ontario.

| **Variable** | **Description** |
| --- | --- |
| **Outcome** | |
| Total LD case counts | Number of confirmed and probable LD cases reported per dissemination area.  Confirmed LD is defined as:   1. Clinical evidence of illness with laboratory confirmed *B. burgdorferi* infection via isolation of bacterium or DNA from a clinical specimen 2. Clinical evidence of illness with two-tiered serological testing confirmation and history of residence/travel to a known LD risk area   Probable LD is defined as:   1. Clinical evidence of illness with two-tiered serologic testing confirmation but without history of residence/travel to a known LD risk area 2. Physician-diagnosed erythema migrans without laboratory evidence but history of residence/travel to a known LD risk area. |
| **Predictors** | |
| Total ticks | Number of publicly submitted *I. scapularis* adults, nymphs, and larva per dissemination area. |
| Total nymphs | Number of publicly submitted *I. scapularis* nymphs per dissemination area. |
| Total *B. burgdorferi* | Number of publicly submitted *I. scap*ularis adults, nymphs, and larva that tested positive for *B. burgdorferi* per dissemination area. |
| Treed land | Proportion of mixed treed land cover (i.e., deciduous, coniferous, mixed forest, shrubs) per dissemination area. |
| Infrastructure | Proportion of infrastructure, roads, and built-up land cover per dissemination area. |
| Walkability score | Score (1-5) is a measure of k-medians cluster of connectivity (3-way intersections), density (dwelling density), and destinations (points of interest) per dissemination area. |
| Income median | Median income range per dissemination area. |
| Commute to work | Categorized measure of duration of commute to work (i.e., less than 15min, 15-29min, 30-44min, 45-59min, over 60min) per dissemination area. |
| Language knowledge | Categorized measure of language knowledge (ie., English only, French only, both English and French, neither English nor French) per dissemination area. |
| Population density | Average population density per dissemination area. |
| Residential instability | Measure refers to area-level concentrations of people who experience high rates of family or housing instability. The indicators included are: proportion of the population living alone, proportion of the population who are not youth (age 5-15), average number of persons per dwelling, proportion of dwellings that are apartment buildings, proportion of the population who are single/divorced/widowed, proportion of dwellings that are not owned, proportion of the population who moved during the past 5 years. |
| Material deprivation | Measure is closely connected to poverty, and it refers to inability for individuals and communities to access and attain basic material needs. The indicators included are proportion of the population aged 20+ without a high-school diploma, proportion of families who are lone parent families, proportion of total income from government transfer payments for population aged 15+, proportion of the population aged 15+ who are unemployed, proportion of the population considered low-income, proportion of households living in dwellings that are in need of major repair. |
| Dependency | Measure refers to area-level concentrations of people who do not have income from employment. Indicators included are proportion of the population who are aged 65 and older, dependency ratio (total population 0-14 and 65+ / total population 15-64), proportion of the population not participating in labour force (aged 15+). |
| Ethnic concentration | Measure refers to high area-level concentrations of people who are recent immigrants and/or people belonging to a ‘visible minority’ group (defined by Statistics Canada as “persons, other than indigenous peoples, who are non-Caucasian in race or non-white in colour”, consisting mainly of the following groups: South Asian, Chinese, Black, Filipino, Arab, Latin American, Southeast Asian, West Asian, Korean and Japanese). Indicators included are proportion of the population who are recent immigrants (arrived in the past 5 years), proportion of the population who self-identify as a visible minority. |
