# Supplementary material for: Spatiotemporal trends and socioecological factors associated with Lyme disease in eastern Ontario, Canada from 2010–2017
Source: BMC Public Health. 2022 Apr 13;22:736. doi: 10.1186/s12889-022-13167-z (PMC9006558; doi:10.1186/s12889-022-13167-z)
Supplement: Supplementary file 2 — Additional file 2. Characteristics of Lyme disease cases and tick submitters with weighted versus unweighted tick exposure location. [file 12889_2022_13167_MOESM2_ESM.docx]

Additional file 2. Characteristics of Lyme disease cases and tick submitters with weighted versus unweighted tick exposure location.

|  | **LYME DISEASE CASES** | | | **TICK SUBMISSIONS** | | |
| --- | --- | --- | --- | --- | --- | --- |
|  | Unweighted  N=592 | Weighted  N=608 | P value | Unweighted  N=1,684 | Weighted  N=4,835 | P value* |
| Age, y ± SD | 48.2 ± 21.0 | 47.7 ± 21.3 | 0.6884 | - | - | - |
| Sex |  |  |  |  |  |  |
| F | 241 | 253 | .5893 | - | - | - |
| M | 348 | 355 | .7918 | - | - | - |
| Unknown | 3 | 0 | - | - | - | - |
| Residence |  |  |  |  |  |  |
| EOH | 70 | 1 | <.0001 | 188 | 439 | <.0001 |
| OTT | 242 | 67 | <.0001 | 585 | 890 | <.0001 |
| LGL | 235 | 188 | .0223 | 478 | 2235 | <.0001 |
| KFL | 45 | 352 | <.0001 | 433 | 1271 | <.0001 |
| Year |  |  |  |  |  |  |
| 2010 | 13 | 2 | .0045 | 10 | 4 | .1088 |
| 2011 | 20 | 16 | .5050 | 235 | 1008 | <.0001 |
| 2012 | 43 | 11 | <.0001 | 324 | 1065 | <.0001 |
| 2013 | 74 | 22 | <.0001 | 437 | 1306 | <.0001 |
| 2014 | 61 | 40 | .0367 | 159 | 590 | <.0001 |
| 2015 | 91 | 113 | .1235 | 51 | 123 | <.0001 |
| 2016 | 69 | 93 | .0593 | 174 | 246 | .0004 |
| 2017 | 221 | 311 | <.0001 | 294 | 493 | <.0001 |

SD: Standard deviation; M: Males; F: Females; EOH: Eastern Ontario Health Unit; OTT: City of Ottawa Health Unit; LGL: Leeds, Grenville, and Lanark Health Unit; KFL: Kingston, Frontenac, Lennox and Addington Health Unit.

* Chi-Square test, Fisher’s exact test, and two-sided t test as appropriate.
