# Supplementary figures and images for: Spatiotemporal trends and socioecological factors associated with Lyme disease in eastern Ontario, Canada from 2010–2017
Source: BMC Public Health. 2022 Apr 13;22:736. doi: 10.1186/s12889-022-13167-z (PMC9006558; doi:10.1186/s12889-022-13167-z)

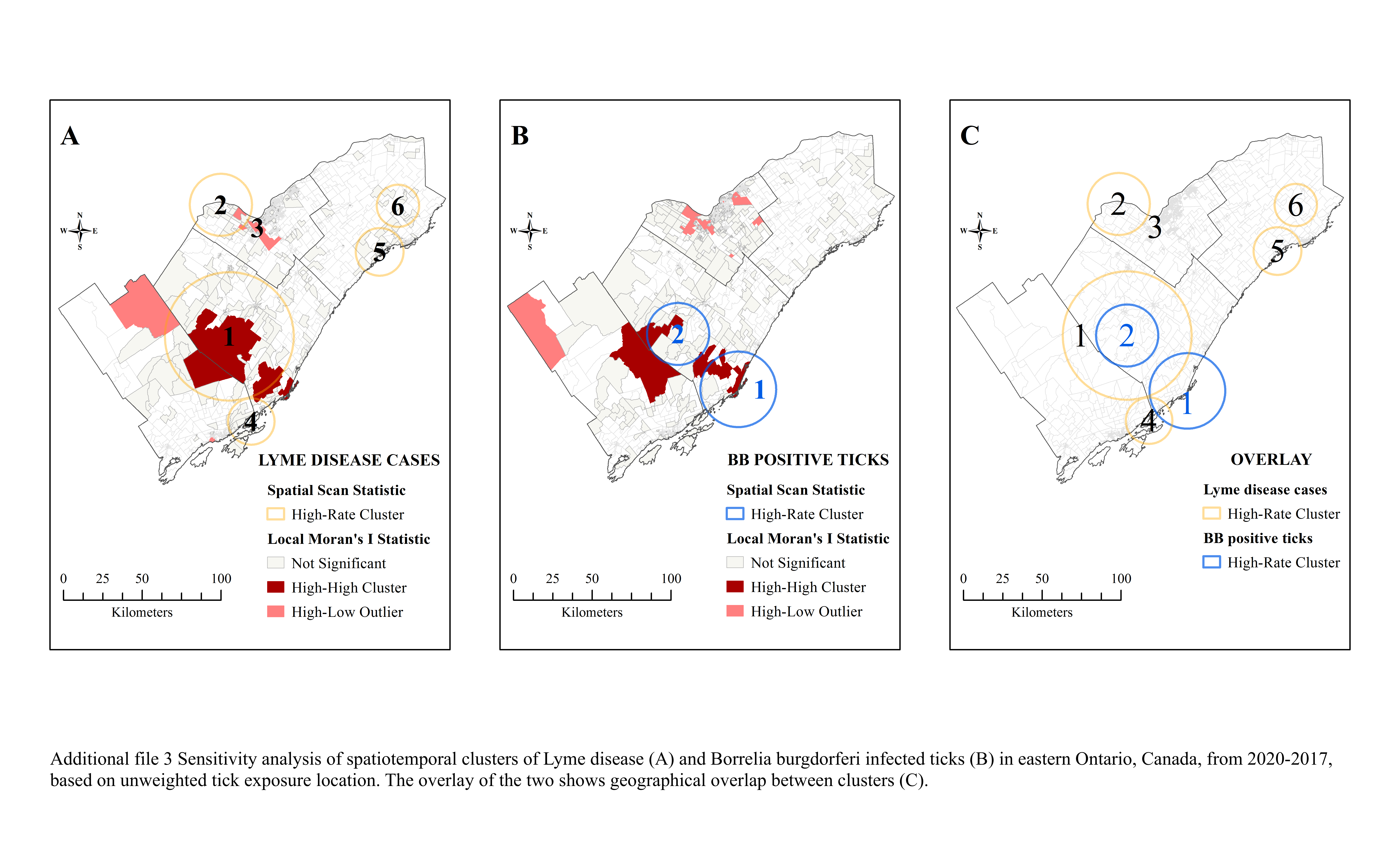

Supplement: Supplementary file 3 — Additional file 3. Sensitivity analysis of spatiotemporal clusters of Lyme disease (A) and Borrelia burgdorferi infected ticks (B) based on unweighted tick exposure location. The overlay of the two shows geographical overlap between clusters (C). [file 12889_2022_13167_MOESM3_ESM.png]
