# Supplementary material for: Spatiotemporal trends and socioecological factors associated with Lyme disease in eastern Ontario, Canada from 2010–2017
Source: BMC Public Health. 2022 Apr 13;22:736. doi: 10.1186/s12889-022-13167-z (PMC9006558; doi:10.1186/s12889-022-13167-z)
Supplement: Supplementary file 4 — Additional file 4. Negative binomial regression model showing socioecological factors associated with Lyme disease case counts in eastern Ontario, Canada from 2010-2017 based on sensitivity analysis dataset with unweighted tick exposures. [file 12889_2022_13167_MOESM4_ESM.docx]

Additional file 4. Negative binomial regression model showing socioecological factors associated with Lyme disease case counts in eastern Ontario, Canada from 2010-2017 based on sensitivity analysis dataset with unweighted tick exposures.

| Parameter | RR | 95% CI | | P value |
| --- | --- | --- | --- | --- |
| Intercept | 0.0514 | 0.0051 | 0.5163 | 0.0117 |
| *B. burgdorferi-*positive ticks | 1.1647 | 1.1082 | 1.2240 | <.0001 |
| Walk score 1 | 1.0000 | - | - | - |
| Walk score 2 | 0.4467 | 0.2861 | 0.6975 | 0.0004 |
| Walk score 3 | 0.5384 | 0.2908 | 0.9967 | 0.0488 |
| Walk score 4 | 0.2942 | 0.0578 | 1.4986 | 0.1408 |
| Walk score 5 | 0.2684 | 0.0244 | 2.9467 | 0.2820 |
| Proportion treed | 1.0220 | 1.0130 | 1.0311 | 0.0001 |
| Residential instability | 1.4384 | 1.1743 | 1.7620 | 0.0004 |
| Ethnic concentration | 0.5688 | 0.4022 | 0.8044 | 0.0014 |
| Log population | 1.4148 | 1.0078 | 1.9862 | 0.0450 |

RR: relative risk, CI: confidence intervals.
